# Whitefly HES1 binds to the intergenic region of *Tomato yellow leaf curl China viru*s and promotes viral gene transcription

**DOI:** 10.1016/j.virol.2020.01.009

**Published:** 2020-03

**Authors:** Yu-Meng Wang, Ya-Zhou He, Xin-Tong Ye, Wen-Ze He, Shu-Sheng Liu, Xiao-Wei Wang

**Affiliations:** Ministry of Agriculture Key Laboratory of Molecular Biology of Crops Pathogens and Insects, Institute of Insect Sciences, Zhejiang University, Hangzhou, China

**Keywords:** *Tomato yellow leaf curl China virus*, Intergenic region, *Bemisia tabaci*, HES1, Viral gene transcription

## Abstract

Intergenic region of begomovirus genome is vital to virus replication and viral gene transcription in plants. Previous studies have reported that *Tomato yellow leaf curl China virus* (TYLCCNV), a begomovirus, is able to accumulate and transcribe in its whitefly vector. However, the viral and host components that participate in begomovirus transcription in whiteflies are hitherto unknown. Using a yeast one-hybrid system, we identified >50 whitefly proteins that interacted with TYLCCNV intergenic region. Dual luciferase analysis revealed that one of the identified proteins, the hairy and enhancer of split homolog-1 (HES1), specifically bound to CACGTG motif in TYLCCNV intergenic region. Silencing *HES1* decreased viral transcription, accumulation and transmission. These results demonstrate that the interactions between whitefly proteins and the intergenic region of TYLCCNV may contribute to viral transcription in the whitefly vector. Our findings offer valuable clues for the research and development of novel strategies to interfere with begomovirus transmission.

## 1 Introduction

Most plant viruses are transmitted by hemipteroid insects including aphids, whiteflies, leafhoppers, planthoppers, and thrips (Hogenhout et al., 2008). According to the characteristics of their transmission by insect vectors, insect-borne plant viruses are classified into two groups, namely non-circulative and circulative (Gray et al., 2014; Dader et al., 2017). While non-circulative viruses are retained by the vector mainly in/on the stylet (food canal) or foregut (Ng and Falk, 2006), circulative viruses move through the insect vector, from the gut lumen into the hemolymph or other tissues and ultimately into the salivary glands (Jia et al., 2018). The latter category is further classified into circulative propagative and circulative non-propagative. Accumulation and replication of plant viruses in insect vectors involve complex interactions between viruses and insect vectors, but the underlying mechanism is still largely unknown. Identification of putative viral and vector components that participate in viral accumulation or replication could profoundly advance our understanding of the nature of virus-vector interactions and provide clues to the development of novel control strategies against plant viruses.

Geminiviruses, characterized by their twin icosahedral capsids and circular single-stranded DNA genome, have caused substantial losses to the production of many economically important crops worldwide (Legg and Fauquet, 2004; Sattar et al., 2013; Shepherd et al., 2010). They have small DNA genomes consisting of one (monopartite) or two (bipartite) DNA components that encode 5–7 proteins involved in viral replication, movement, transmission and pathogenesis (Hanley-Bowdoin et al., 2013; Seal et al., 2006). In the genomes of geminiviruses, alongside the coding sequences, there is a intergenic region of *ca.* 300 nt that contains a conserved hairpin structure and viral promoters, serving as binding sites for viral replication initiator protein (Rep), host RNA polymerase II, and other host factors during *in planta* virus replication (Hanley-Bowdoin et al., 1999). Site-directed mutagenesis of viral intergenic region reduced viral genes transcription to various extents and even influenced virus replication, indicating that the intergenic region of geminivirus is crucial for viral transcription and replication in plants (Eagle and Hanley-Bowdoin, 1997; Hanley-Bowdoin et al., 1999; Orozco and Hanley-Bowdoin, 1996).

*Begomovirus* is the largest genus of the family *Geminiviridae* and currently contains 409 accepted species (https://talk.ictvonline.org/as accessed on 14 October 2019). Begomoviruses are exclusively transmitted by whiteflies of the *Bemisia tabaci* cryptic species complex in a circulative manner (Navas-Castillo et al., 2011; Czosnek et al., 2017). These viruses are acquired from plant sap ingested by whiteflies and first overcome the gut barriers to get into the hemolymph (Pan et al., 2017, 2018a, 2018b; Guo et al., 2018; Xia et al., 2018; Zhao et al., 2019). Then some of these viruses are able to translocate to the salivary gland and finally are secreted into new host plants (Wei et al., 2014). Viruses may also get into the female ovary to be transmitted to whitefly offspring (Wei et al., 2017; Guo et al., 2019). Interestingly, although begomoviruses are generally believed not to replicate in their whitefly vectors, several studies have reported active gene transcription of some begomoviruses in whiteflies (Czosnek et al., 2001; Wang et al., 2016, 2017). Sinisterra et al. (2005) found that the transcripts of TYLCV in whiteflies increased after transfer of whiteflies to cotton, a TYLCV non-host plant, following acquisition from virus-infected plants. In contrast, the gene transcripts of *Tomato mottle virus* (ToMoV) rapidly become undetectable in whiteflies following transfer from infected tomato to cotton, suggesting specific gene transcription of some begomoviruses in the whitefly vector. Recently, active gene transcription of *Tomato yellow leaf curl China virus* (TYLCCNV), which is closely related to TYLCV, was observed in viruliferous whiteflies (Wang et al., 2017). Moreover, when whiteflies that had fed on virus-infected plants for 6 h were transferred to feed on cotton, a non-host plant of TYLCCNV, the amount of TYLCCNV DNA and coat protein (CP) in whiteflies increased in the first two days (Wang et al., 2016). These observations indicate that TYLCCNV may be able to transcript and replicate in whiteflies.

Previous studies showed that host plant transcription factors bind to the intergenic region of begomoviruses to regulate viral transcription and replication in plants (Eagle et al., 1994; Groning et al., 1994; Lazarowitz, 1992; Orozco et al., 1998; Sunter and Bisaro, 1997; Eagle and Hanley-Bowdoin, 1997). We thus speculated that some whitefly proteins may participate in viral transcription and accumulation in whiteflies by interacting with TYLCCNV intergenic region. To test this hypothesis, first we used a yeast one-hybrid system to screen whitefly proteins that may interact with the intergenic region of TYLCCNV, and identified >50 putative proteins. Next, we experimentally examined the interaction of the hairy and enhancer of split homolog-1 (HES1) transcription factor, one of the whitefly proteins identified, with the intergenic region of TYLCCNV. Finally, we tested the roles of HES1 in viral transcription, accumulation and transmission. Our results suggest that the whitefly transcription factor HES1 may be involved in the accumulation of begomovirus in whiteflies via binding to the intergenic region of the virus and regulating its gene transcription.

## 2 Material and methods

### 2.1 Insects, virus and plants

A population of Middle East Asia Minor 1 (MEAM1) (mitochondrial cytochrome oxidase I GenBank accession no. GQ332577), a putative species of the *B. tabaci* complex was used in this study. Whiteflies were reared on cotton plants (*Gossypium hirsutum* L. cv. Zhemian 1793) in insect-proof cages at 26 °C (±1 °C) under a photoperiod of 14:10 h (light/dark) and a relative humidity of 60% (±10%). The purity of the population was monitored every three generations by amplifying and sequencing the mitochondrial cytochrome oxidase I gene, which has been widely used to differentiate *B. tabaci* genetic groups (Liu et al., 2012). Infectious clones of TYLCCNV isolate Y10 (GenBank accession no. AJ319675) in conjunction with its associated beta-satellite (TYLCCNV beta-satellite, GenBank accession no. AJ421621) were agro-inoculated into 3–4 true leaf stage tobacco (*Nicotiana tabacum* cv. NC89) plants, and the plants were used approximately 3–4 weeks post virus inoculation. All plants were grown in insect-proof greenhouses under controlled temperature at 25 ± 3 °C and natural lighting.

### 2.2 Yeast one-hybrid (Y1H) assay

Y1H assay was performed using the Matchmaker Gold Yeast One-Hybrid Library Screening System and Yeastmaker Yeast Transformation System (Clontech) as described in the manufacturer's instructions. A cDNA library of *B. tabaci* was constructed in the prey plasmid, SfiⅠ-digested pGADT7, using the SMART cDNA library construction kit (Clontech). The bait vector was constructed by fusing the three tandem copies of the left part (NVL) (5′-TAGAAGTATTAAATGTTAATACGGTAAACCACAGGTGTATATTTATTCTGGGGTCTGTGGCTAACGTATCGGTTCAAACTCTCTGTGGTTAACTGGTCAGT-3′) or the right part (NVR) (5′-TTAAAGTGGTCCCCGCAGACACGTGTGTCCAATCTTGGCCACTCCTCAAAGCTTAATTGTTAAATGGTCCCCTATAAAACTTAGCGCCCAAGTATTCACGTTAAGC-3′) of the TYLCCNV intergenic region into pAbAi with *SacI* and *SalI*, respectively.

To confirm that the plasmid was integrated correctly, we used a colony PCR analysis with the Matchmaker Insert Check PCR Mix (Clotech). After screening different concentrations of Aureobasidin A (AbA) to suppress basal expression of bait constructs, we found no background growth of yeast when AbA concentration was 200 ng/ml for the left part of intergenic region and 250 ng/ml for the right part. cDNA library was then transformed into the yeast containing integrated parts of intergenic region. Plasmids from the positive clones were recovered, and transformed into *Escherichia coli* strain DH5α and sequenced thereafter. The sequences obtained were then used in a BLAST search against NCBI database (http://blast.st-va.ncbi.nlm.nih.gov/Blast.cgi). Gene Ontology (GO) annotations of molecular function were assigned according to the Blast2go database (http://www.blast2go.com). The proteins were determined using the ortholog annotation from *Drosophila melanogaster* in FlyBase (http://flybase.org/). GO annotation results were visualized and plotted using Web Gene Ontology Annotation Plot (http://wego.genomics.org.cn/).

### 2.3 Double-stranded (dsRNA) synthesis

DsRNA was synthesized using the T7 High Yield RNA Transcription kit (Vazyme), following the manufacturer's instructions. Briefly, the DNA template for dsRNA synthesis was amplified with primers containing the T7 RNA polymerase promoter at both ends (Table S1), and the purified DNA template was then used to synthesize dsRNAs. Subsequently, the synthesized dsRNA was purified via phenol-chloroform precipitation and re-suspended in nuclease-free water. The concentration of dsRNA was determined with a NanoDrop 2000 (Thermo Fisher Scientific). At the same time, the quality and size of the dsRNAs were further verified with electrophoresis in a 2% agarose gel.

### 2.4 Gene silencing by oral ingestion of dsRNA

For gene silencing, newly emerged whiteflies were first given a 6 h acquisition access period (AAP) on TYLCCNV-infected plants, and then were collected for dsRNAs feeding. For oral ingestion of dsRNA, feeding chambers were made using glass tubes (diameter: 1.5 cm, length: 10 cm). One end of each tube was covered with double layers of parafilm that was filled with a 15% (wt/vol) sucrose diet containing dsRNA of a concentration 250 ng/μl. Approximately 100 adult whiteflies were placed in each feeding chamber and left there to feed for 48 h. Thereafter, from each feeding chamber, 20 female adults were collected and their total DNA was extracted to examine the abundance of viral DNA, and 30 female adults were collected to extract their total RNA for observing HES1 and TYLCCNV-CP mRNA level. Approximately 200 adults of mixed-sex were collected to extract their total protein for western blot assay. To assess the effect of *dsHES1* treatment on the abundance of TYLCCNV at different time points in whiteflies, newly emerged whiteflies were collected and placed on TYLCCNV-infected tobacco plants to feed 6 h and were then collected to feed on the dsRNA diet in feeding chambers (as described above) for 48 h. After that, the viruliferous whiteflies were transferred to the next batch of feeding chambers to feed on the 15% (wt/vol) sucrose diet without dsRNA for 96 h, and whiteflies were collected at 0, 12, 24, 48, 96 and 144 h after virus acquisition.

### 2.5 Quantification of virus and analysis of gene transcription

For viral DNA quantification, groups of 20 whiteflies each were ground in 40 μL of ice-cold lysis buffer (50 mM Tris-HCl with pH at 8.4, 0.2% gelatin, 0.45% Tween 20, 0.45% Nonidet P-40, and 60 mg/l proteinase K) and were incubated at 65 °C for 2 h, and then at 100 °C for 10 min. The supernatants were kept at −20 °C. For analysis of gene transcription, total RNA was isolated from groups of 30 whiteflies each using TRIzol reagent (Ambion), and then cDNAs were generated using the PrimeScript RT reagent kit with gDNA Eraser (TaKaRa). Quantitative PCR (qPCR) was performed using CFX Connect Real-Time PCR System (Bio-Rad) with SYBR Premix Ex TaqTM II (TaKaRa) and the primers are shown in Table S1. For each reaction, 0.8 μL of each primer (10 mM), 6.4 μL of nuclease-free water, 2 μL of sample and 10 μL of SYBR Premix Ex Taq were added to a total volume of 20 μL. The qPCR protocol was 95 °C for 30 s, followed by 40 cycles of at 95 °C for 5 s and 60 °C for 30 s. A negative control (using nuclease-free water instead of sample) was included throughout the experiments to detect contamination and to determine the degree of dimer formation.

### 2.6 Cell culture, plasmid and transfection

*Drosophila* Schneider S2 cells were maintained at 27 °C in *Drosophila* Serum-Free Medium supplemented with 10% heat-inactivated fetal bovine serum (Gibco). pAc5.1/V5-HisB (Invitrogen) was used for expression of full length HES1 protein. The full-length 115 bp right part of TYLCCNV intergenic region, 1-45bp (NV-R1), 46-80bp (NV-R2), 81-115bp (NV-R3) and NV-R1-M were chemically synthesized and sub-cloned into reporter vector pGL3-Basic vector (Promega). The constructions were confirmed by sequencing. Twelve hours before transfection, cells were seeded in a 24-well plate at 2 × 10^5^ cells per well. Transfections with expression plasmids, reporter plasmid and Renilla luciferase plasmid were conducted using Lipofectamine 3000 (Invitrogen). At 72 h post-transfection, the cells were harvested and processed with Dual-Luciferase Reporter Assay Kit (Vazyme) according to the manufacturer's protocol. The luciferase activities were measured using FlexStation-3 microplate reader (Molecular Devices). The activities of firefly luciferase were normalized to that of Renilla luciferase. All experiments were carried out in triplicate.

### 2.7 Western blot

To detect TYLCCNV CP in whiteflies, samples of 200 whiteflies each were prepared using radioimmunoprecipitation assay buffer with proteinase inhibitors. Protein samples were separated using 4–20% sodium dodecyl sulfate-polyacrylamide gel electrophoresis, and then transferred onto PVDF membrane. The membrane was blocked with 5% milk in TBS (10 mM Tris HCl, 150 mM sodium chloride, pH 7.5), and then incubated with appropriate primary antibodies. After incubation with horseradish peroxidase (HRP)-conjugated secondary antibodies, blots were visualized with the ECL Plus Detection System (Bio-Rad). Antibodies to TYLCCNV CP (IC4) were kindly provided by Professor Jianxiang Wu, the Institute of Biotechnology, Zhejiang University. Commercial antibodies to ACTIN (beta-actin) were purchased from EARTHOX life sciences.

To detect the expression of HES1 in S2 cells, cells were washed twice using PBS and then re-suspended with lysis buffer (50 mM Tris-HCl, 150 mM NaCl, 1% Nonidet P-40, pH 7.8). After centrifugation, the supernatant was boiled in PAGE buffer for 5 min. The presence of target proteins was verified using western blotting with an anti-His monoclonal antibodies (Abcam).

### 2.8 Transmission of virus to plants by whiteflies

Following virus acquisition and gene silencing, TYLCCV transmission assay was performed. Viruliferous whiteflies were collected as groups of 10 (female/male = 1:1) each, and tobacco seedlings at the 3–4 true-leaf stage (*ca.* 3 weeks after sowing) were each inoculated with one group of 10 whiteflies, which were placed on the underside of the top second leaf of a plant to feed and inoculate for 3 days, using a leaf clip cage (Pan et al., 2018a). For each treatment, three replicates of 10 plants each were conducted. The plants were then sprayed with imidacloprid at a concentration of 20 mg/L to kill all the whitefly adults and eggs, and maintained until disease symptoms were observed. Virus infection of test plants was determined by symptom inspection and PCR.

### 2.9 Statistical analysis

The relative abundance of viral DNA and relative level of gene expression were calculated using the 2^−Δ Ct^ method as normalized to the level of *B. tabaci β-actin* gene. For comparison of virus transmission efficiency, percentage data were arcsine square root transformed. The independent-sample *t*-test was performed to compare two means, and one-way ANOVA followed by least significant difference (LSD) test was applied for multiple comparisons. All analyses were performed using SPSS 13.0.

## 3 Results

### 3.1 Construction of the bait plasmid and bait yeast strain

**Fig. 1:**
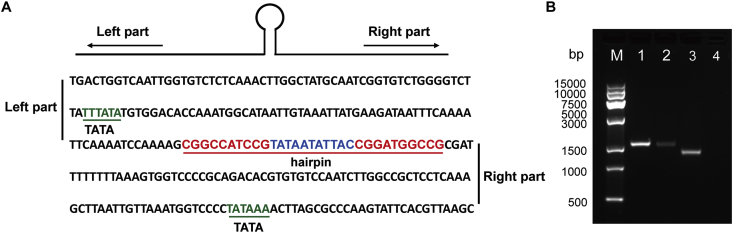
**The sequence of TYLCCNV intergenic region and verification of bait integration in yeast strain.** (A) The virion-sense DNA sequence of TYLCCNV intergenic region corresponding to positions 2593 to 127 in TYLCCNV genome is shown. The hairpin structure and TATA box are underlined. The intergenic region was divided into left part and right part according to the hairpin structure and inserted into yeast one-hybrid vector pAbAi, respectively. (B) Amplification of integrated sequences in different yeast strains. M, DNA Marker; 1, Y1HGold (pNVL-AbAi) yeast strain; 2, Y1HGold (pNVR-AbAi); 3, Y1HGold (p53-AbAi, positive control); 4, Y1HGold (negative control).

The transcription of geminivirus genes is bidirectional, resulting in mRNAs that correspond to both the virion-sense and complementary-sense open reading frames (Frischmuth et al., 1991; Sunter et al., 1989). Considering the minimal sequence required for activation of viral genes (Ramos et al., 2004; Sunter and Bisaro, 2003), the sequences in the upstream (left part) and downstream (right part) of the hairpin structure from TYLCCNV intergenic region were selected to represent the viral promoters (Fig. 1A). The bait vectors (pAbAi) were constructed using three tandem copies of these two parts and then were linearized and integrated into the genome of yeast (Y1H Gold). As shown in Fig. 1B, the PCR products of yeast strain Y1HGold (pNVL-AbAi) and Y1HGold (pNVR-AbAi) were 1.653 kb and 1.668 kb in length, while the PCR products of Y1HGold (p53-AbAi) (positive control) were 1.4 kb and no amplification was found in the negative control.

### 3.2 Screening of whitefly proteins that interact with TYLCCNV intergenic region by Y1H

We screened the whitefly cDNA library and confirmed the reporter phenotype by re-streaking onto fresh selective media. In total, we identified 32 proteins interacting with the left part of intergenic region (Table 1) and 20 proteins with the right part (Table 2). All of the identified protein sequences matched whitefly proteins, except one protein that matched to whitefly bacterial symbiont *Portiera aleyrodidarum*. According to the GO annotation analysis, most of the isolated proteins have binding and catalytic activity and participate in cellular and metabolic processes (Fig. S1).

**Table 1:** BLAST results of putative proteins that interacts with the left part of TYLCCNV intergenic region.

No.	Genbank accession No.	Protein
**1**	**XP_018905833.1**	**phosphatidylethanolamine-binding protein 2-like (*PEBP 2-like*) isoform X1 [*Bemisia tabaci*]**
**2**	**XP_018906550.1**	**late endosomal/lysosomal adaptor and MAPK and MTOR activator 4 (LAMTOR4) [*Bemisia tabaci*]**
**3**	**XP_018916034.1**	**DDB1- and CUL4-associated factor 6-like (*DCAF6-like*) [*Bemisia tabaci*]**
**4**	**WP_014895075.1**	**ATP-dependent protease ATPase subunit (*HslU*) [Candidatus *Portiera aleyrodidarum*]**
**5**	**XP_018896692.1**	**nucleolar protein 11 (*NOL 11*) [*Bemisia tabaci*]**
**6**	**XP_018910249.1**	**trafficking kinesin-binding protein (*TRAK*) milt isoform X4 [*Bemisia tabaci*]**
**7**	**XP_018914581.1**	**transcriptional adapter 3-A (*ADA 3-A*) [*Bemisia tabaci*]**
8	XP_018910563.1	ubiquitin carboxyl-terminal hydrolase (*FAM188A*)homolog [*Bemisia tabaci*]
9	XP_018915661.1	V-type proton ATPase (*V-ATPase*) subunit C [*Bemisia tabaci*]
10	XP_018906084.1	checkpoint protein HUS1 (*HUS1*) [*Bemisia tabaci*]
11	XP_018911370.1	dehydrogenase/reductase (*SDR family*) member 7 [*Bemisia tabaci*]
12	XP_018902726.1	max dimerization protein 1-like (*MAD1*) isoform X1 [*Bemisia tabaci*]
13	XP_018902781.1	DNA-directed RNA polymerase I subunit (*RPA2*) [*Bemisia tabaci*]
14	XP_018897419.1	prefoldin subunit 6 [*Bemisia tabaci*]
15	XP_018916029.1	proteoglycan Cow isoform X2 [*Bemisia tabaci*]
16	XP_018906471.1	mediator of RNA polymerase II transcription subunit 22 (*MED22*) [*Bemisia tabaci*]
17	XP_018907204.1	longitudinals lacking protein (*LOLA*), isoforms H/M/V-like isoform X3 [*Bemisia tabaci*]
18	XP_018909301.1	eukaryotic translation initiation factor 3 (*eIF3*) subunit G-like [*Bemisia tabaci*]
19	XP_018913632.1	phosphatidylinositol 4-phosphate 5-kinase type-1 alpha (*PIP5K1A*) isoform X7 [*Bemisia tabaci*]
20	XP_018907621.1	ribosomal protein S6 kinase beta-1-like (*RPS6KB1*) isoform X1 [*Bemisia tabaci*]
21	XP_018907768.1	60S ribosomal protein L13a (*RPL13a*) [*Bemisia tabaci*]
22	XP_018910162.1	28S ribosomal protein S5 (*RPS5*), mitochondrial [*Bemisia tabaci*]
23	XP_018911168.1	complex I intermediate-associated protein 30 (*CIA30*), mitochondrial [*Bemisia tabaci*]
24	XP_018900645.1	phosducin-like protein (*PhLP*) [*Bemisia tabaci*]
25	XP_018916856.1	3-ketoacyl-CoA thiolase (*KAT*), mitochondrial [*Bemisia tabaci*]
26	XP_018907134.1	serine/arginine repetitive matrix protein 2-like isoform X2 [*Bemisia tabaci*]
27	XP_018896979.1	39S ribosomal protein L19 (*RPL19*), mitochondrial [*Bemisia tabaci*]
28	XP_018917530.1	apyrase-like [*Bemisia tabaci*]
29	XP_018903751.1	NADH dehydrogenase [ubiquinone] 1 alpha subcomplex subunit 9 (*NDUFA9*), mitochondrial [*Bemisia tabaci*]
30	BAX57176.1	phosphate carrier 2 [*Bemisia tabaci*]
31	XP_018917258.1	maltase 2-like [*Bemisia tabaci*]
32	XP_018907264.1	signal recognition particle 54 kDa protein (*SRP54*) [*Bemisia tabaci*]

Note:Proteins 1–19 were selected to confirm the interactions with the left part of TYLCCNV intergenic region in yeast one-hybrid system, and proteins 1–7 were experimentally examined for its interaction.

**Table 2:** BLAST results of putative proteins that interact with the right part of TYLCCNV intergenic region.

No.	Genbank accession No.	Protein
**1**	**XP_018914543.1**	**max-like protein X [*Bemisia tabaci*]**
**2**	**XP_018910361.1**	**60S ribosomal protein L18a (*RPL18a*) [*Bemisia tabaci*]**
**3**	**XP_018911559.1**	**transcription factor HES-1-like isoform X2 [*Bemisia tabaci*]**
**4**	**XP_018910391.1**	**protein tipE [*Bemisia tabaci*]**
**5**	**XP_018905833.1**	**phosphatidylethanolamine-binding protein 2-like (*PEBP 2-like*) isoform X1 [*Bemisia tabaci*]**
6	XP_018907892.1	ribosomal protein S6 kinase alpha-2 (*RPS6KA2*) isoform X2 [*Bemisia tabaci*]
7	XP_018917343.1	fumarate hydratase, mitochondrial-like [*Bemisia tabaci*]
8	XP_018909408.1	ribonuclease H2 subunit A [*Bemisia tabaci*]
9	XP_018901241.1	nucleoplasmin-like protein isoform X1 [*Bemisia tabaci*]
10	XP_018912337.1	transcription initiation factor TFIID subunit 12 [*Bemisia tabaci*]
11	XP_018903751.1	NADH dehydrogenase [ubiquinone] 1 alpha subcomplex subunit 9 (*NDUFA9*), mitochondrial [*Bemisia tabaci*]
12	XP_018906539.1	probable chitinase 3 [*Bemisia tabaci*]
13	XP_018912518.1	RWD domain-containing protein 4 [*Bemisia tabaci*]
14	XP_018909031.1	60 kDa heat shock protein (*HSP60*), mitochondrial [*Bemisia tabaci*]
15	XP_018900567.1	probable ATP-dependent RNA helicase DDX28 [*Bemisia tabaci*]
16	XP_018917833.1	BTB and MATH domain-containing protein 43-like [*Bemisia tabaci*]
17	XP_018911340.1	probable E3 ubiquitin-protein ligase HERC1 [*Bemisia tabaci*]
18	XP_018909032.1	10 kDa heat shock protein (*HSP10)*, mitochondrial [*Bemisia tabaci*]
19	XP_018899896.1	la-related protein 7 [*Bemisia tabaci*]
20	XP_018909988.1	eukaryotic translation initiation factor 4E-like (*eIF4E*) [*Bemisia tabaci*]

Note:Proteins 1–13 were selected to confirm the interactions with the right part of TYLCCNV intergenic region in yeast one-hybrid system, and protein 1-5 were experimentally examined for finally determined to interact with it.

**Fig. 2:**
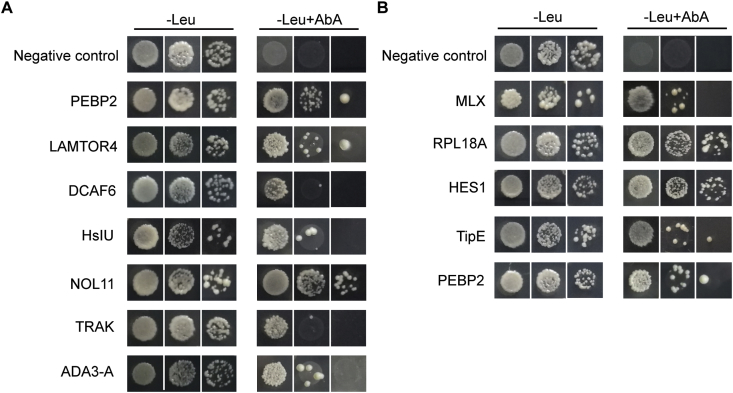
**Verification of interaction between whitefly proteins and TYLCCNV intergenic region using yeast one-hybrid (Y1H) assay.** The concentration of Aureobasidin A (AbA) used in selective medium (-Leu + AbA) was 200 ng/ml (A) and 250 ng/ml (B). Yeast growth on selective medium (-Leu + AbA) was recorded on day 3 as an indicator of protein-DNA interactions. Empty vector of pGAD served as negative control. (A) Proteins interacting with the left part of intergenic region of TYLCCNV. PEBP2, phosphatidylethanolamine-binding protein 2-like. LAMTOR4, late endosomal/lysosomal adaptor and MAPK and MTOR activator 4. DCAF6, DDB1- and CUL4-associated factor 6-like. HsIU, ATP-dependent protease ATPase subunit. NOL11, nucleolar protein 11. TRAK, trafficking kinesin-binding protein. ADA3-A, transcriptional adapter 3-A. (B) Proteins interacting with the right part of intergenic region of TYLCCNV. MLX, max-like protein X. RPL18a, 60S ribosomal protein L18a. HES1, hairy and enhancer of split homolog-1 like.

To confirm the interaction between TYLCCNV intergenic region and the isolated prey proteins, we selected 19 proteins screened from the left part of intergenic region (No. 1–19 in Table 1) and 13 proteins from the right part (No. 1–13 in Table 2) for further analysis. The results showed that seven proteins: phosphatidylethanolamine-binding protein 2-like (PEBP2), late endosomal/lysosomal adaptor and MAPK and MTOR activator 4 (LAMTOR4), DDB1- and CUL4-associated factor 6-like (DCAF6), ATP-dependent protease ATPase subunit (HsIU), nucleolar protein 11 (NOL11), trafficking kinesin-binding protein (TRAK) and transcriptional adapter 3-A (ADA3-A) interacted with the left part of viral intergenic region (Fig. 2A). Five proteins: max-like protein X (MLX), 60S ribosomal protein L18a (RPL18a), hairy and enhancer of split homolog-1 like (HES1), protein tipE and PEBP2 interacted with the right part of viral intergenic region (Fig. 2B) in yeast. Interestingly, PEBP2 interacted with both parts of TYLCCNV intergenic region.

### 3.3 Effect of candidate genes knockdown on viral accumulation in whiteflies

**Fig. 3:**
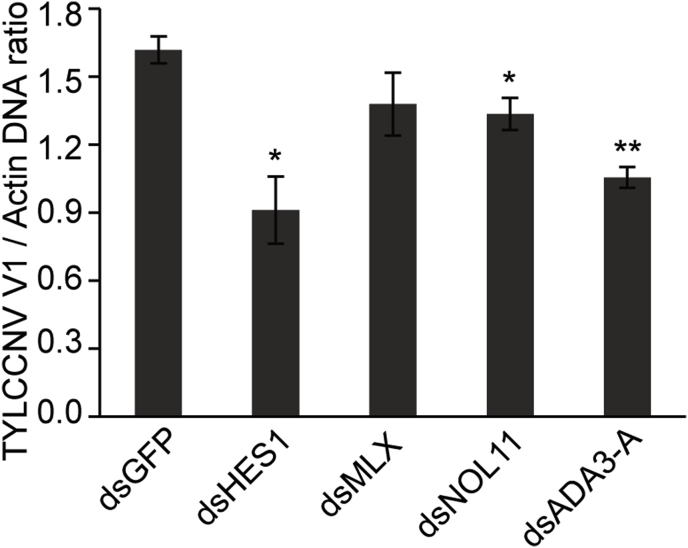
**Effect of knockdown of various candidate genes on virus accumulation in whiteflies.** Quantitative analysis of viral DNA in whiteflies that fed with dsRNAs after a 6 h AAP on TYLCCNV infected tobacco plants. Data are presented as mean ± SEM of three independent experiments and analyzed statistically using independent-simple *t*-test (*, *P* < 0.05; **, *P* < 0.01).

We selected MLX, HES1, NLO11 and ADA3-A for further analysis as these proteins contain a DNA-binding domain and/or have a relatively higher level of interaction with TYLCCNV intergenic region. To investigate the role of these genes in viral accumulation, we suppressed the expression of these genes using RNA interference (RNAi). Whiteflies were firstly fed on a TYLCCNV-infected tobacco for 6 h. Then they were collected randomly and fed on dsRNA corresponding to each of these genes respectively for another 48 h. Whiteflies fed with dsGFP were used as control. Quantitative analysis showed that the amount of TYLCCNV DNA was decreased in dsHES1, dsNOL11 and dsADA3-A treated whiteflies compared with that of the control (Fig. 3).

### 3.4 Characterization of whitefly HES1

Because dsHES1-treated whiteflies showed the largest decrease of virus among the four genes tested, we selected HES1 for further investigation (Fig. 3). The partial *HES1* sequence obtained in screening was searched against the GenBank, and a predicted open reading frame (ORF) of whitefly *HES1* was identified (GenBank accession no. XP_018911559.1). Based on the predicted sequence, the 999 bp ORF of whitefly *HES1* was cloned, which encoded a 332 amino acid protein with a calculated molecular mass of ~36.8 kDa. The *HES1* partial sequence identified from the yeast clones was from nucleotide 159 to 999 of the full length ORF. Domain architecture analysis by SMART server predicted the presence of two conserved function domains of HES factors viz. HLH domain (nucleotides 256–429) and Orange domain (nucleotides 436–591) (Fig. S2A) (Kageyama et al., 2007).

### 3.5 HES1 binds to viral intergenic region specifically

**Fig. 4:**
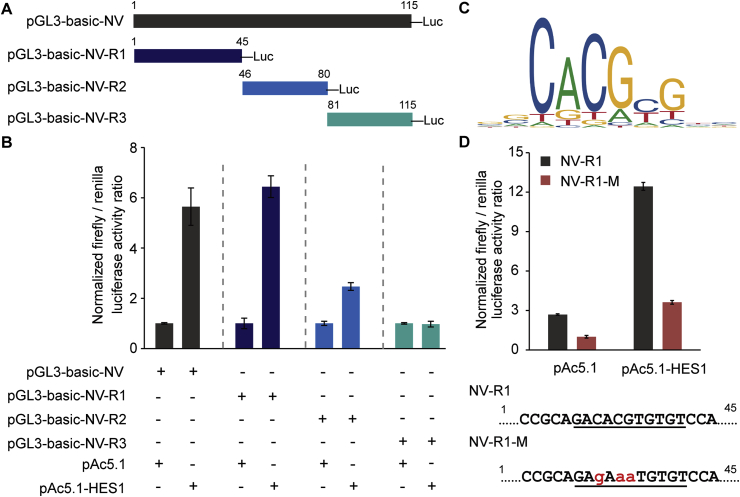
**Identification of HES1 binding sites of TYLCCNV intergenic region.** (A) Schematic diagrams of the constructs used in dual luciferase reporter assay. The full-length (1-115bp), 1-45bp (NV-R1), 46-80bp (NV-R2) and 81-115bp (NV-R3) sequence of the right part of TYLCCNV intergenic region was fused to firefly luciferase gene, respectively. (B) Luciferase report assays after cotransfection of expression vectors pAc5.1 and reporter vectors pGL3-basic into S2 cells. Treatments with empty expression vector served as controls. Firefly luciferase activity was normalized to the *Renilla* luciferase activity. Error bars represent ± SEM (n = 3). (C) The matrix of HES1 in vertebrata database of JASPAR. The sequence logo was downloaded from JASPAR (http://jaspar.genereg.net/) and the matrix ID is MA1099.1. (D) Putative HES1 binding elements in TYLCCNV intergenic region and mutated sequence for experiments are underlined. Cotransfection with wild-type (NV-R1) or mutant (NV-R1-M) reporter vector, together with expression vector was performed. The experiments were undertaken as mentioned above. Error bars represent ± SEM (n = 3).

To examine the role of the interaction between HES1 and the intergenic region in its downstream gene expression, the right part of the intergenic region was cloned into luciferase reporter vector pGL3basic (Fig. 4A). After confirming the successful expression of whitefly HES1 protein in *Drosophila melanogaster* S2 cells by western blot analysis (Fig. S2B), we co-transfected luciferase reporter vector and expression vector pAc5.1 into the cell culture system. Compared with the control (transfection of empty expression vector), HES1 overexpression resulted in a more than 5-fold increase in luciferase activity when a full-length of the right intergenic region was cloned into the reporter vector (Fig. 4B). To pinpoint the specific binding sites of HES1, we divided the right part of intergenic region into three parts and cloned each of them into the reporter vector (Fig. 4A). The dual-luciferase assay revealed an over 6-fold increase of the promoter activity in the presence of NV-R1. In contrast, only a slight or no increase in luciferase activity was observed for NV-R2 and NV-R3, respectively (Fig. 4B). It suggests that NV-R1 likely harbors a potential HES1 binding site. We then scanned the right part of TYLCCNV intergenic region using the matrix of HES1 in vertebrata database of JASPAR. Consistent with the results of dual-luciferase assays, a possible HES1 binding site was found in the NV-R1 (Fig. 4C). Moreover, the mutation of the CACGTG sequence of NV-R1 to GAAATG (NV-R1-M) repressed the reporter activity when supplied with empty expression vector or HES1 fusion vector (Fig. 4D), demonstrating the specific interaction between HES1 and this motif. Taken together, these data prove that the whitefly HES1 specifically binds to the right part of TYLCCNV intergenic region to activate the transcription of its downstream genes.

### 3.6 HES1 contributes to viral transcription and accumulation in whiteflies

**Fig. 5:**
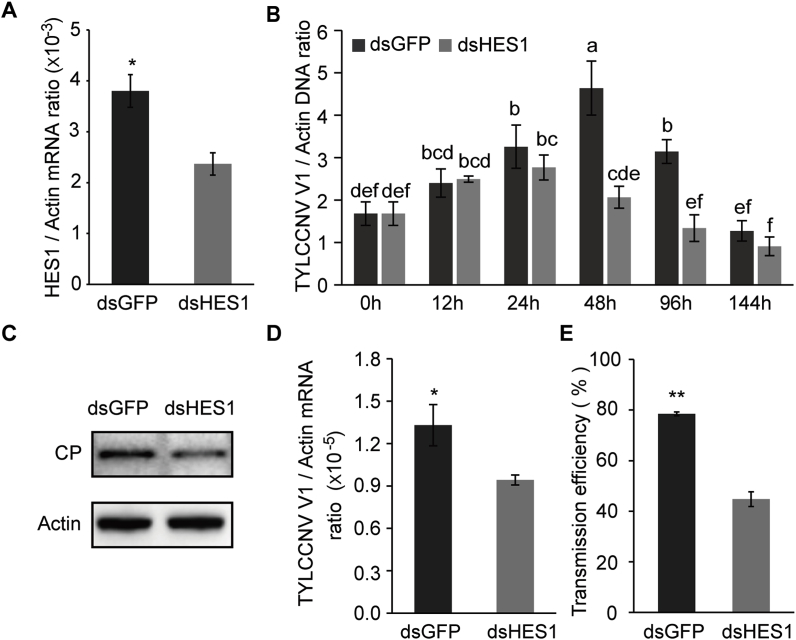
**Effect of silencing HES1 on viral transcription and accumulation in whiteflies.** (A) Quantitative analysis of HES1 mRNA level after feeding with dsRNAs. (B) Quantitative analysis of viral DNA in whiteflies that were collected at 0, 12, 24, 48, 96 and 144 h after a 6 h AAP on TYLCCNV infected tobacco plants. After acquisition, whiteflies were fed with dsRNA for 48 h and then transferred to 15% (wt/vol) sucrose diet for another 96 h. Mean ± SEM of three independent experiments. *P* < 0.05 (one-way ANOVA, LSD test) was taken as significant. Immunoblot analysis of TYLCCNV CP (C) and quantitative analysis of viral V1 mRNA (D) in whiteflies that were fed with dsRNAs after a 6 h AAP on TYLCCNV infected tobacco plants. Actin served as loading control in western blots. (E) Virus transmission efficiency of dsRNAs treated whiteflies following a 6 h AAP on TYLCCNV infected tobacco plants. Plant infection was determined by monitoring disease symptoms and by PCR amplification of viral DNA at 30 days post inoculation. Three replicates were conducted with each containing 10 test plants. (A, D and E). Data are presented as mean ± SEM of three independent experiments and analyzed statistically using independent-simple *t*-test (*, *P* < 0.05; **, *P* < 0.01).

To verify the role of HES1 in TYLCCNV transcription and accumulation in whiteflies, we silenced the expression of *HES1* by RNAi. After a 6 h acquisition on virus-infected plants, whiteflies were fed with dsHES1 or dsGFP for 48 h and then transferred to artificial diet for another 96 h. Compared with dsGFP treatment, HES1 mRNA level decreased by 37% in dsHES1-treated whiteflies (Fig. 5A). Quantitative analysis showed that the abundance of TYLCCNV DNA increased gradually in dsGFP-treated whiteflies, peaked at 48 h and then decreased. The virus abundance in dsHES1-treated whiteflies also increased gradually; however, it peaked at 24 h and then decreased. Moreover, the amount of virus in dsHES1-treated whiteflies was significantly lower than that of dsGFP-treated whiteflies at 48 and 96 h after acquisition (Fig. 5B). The expression level of viral CP encoding gene (*V1*) and the amount of CP in dsHES1-treated whiteflies also decreased at 48 h after acquisition (Fig. 5C and D). Virus transmission assays further showed that the disease prevalence of dsHES1-treated whiteflies inoculated plants was significantly lower than that of the control group (Fig. 5E). By the 30th day, 78.5% of plants in the control group were symptomatic, while only 44.8% in the dsHES1-treated group developed symptoms. Overall, these results suggest that HES1 facilitates viral transcription and accumulation in whiteflies, and in turn promotes virus transmission by insect vectors.

## 4 Discussion

Intergenic region of geminiviruses contains promotors and/or regulatory elements that are essential for viral transcription and replication in plants (Borah et al., 2016; Velten et al., 2005). The minimal sequence required for activation of *Tomato mottle Taino virus* (ToMoTV) *Rep* promoter is located at the left side of its intergenic region and within ~130 nt upstream of the gene transcription start site (Ramos et al., 2004). Moreover, as few as ~63 nt upstream sequence of *Tomato golden mosaic virus* (TGMV) *CP* locating at the right side of its intergenic region is required for *CP* promoter expression (Sunter and Bisaro, 2003). Due to the limited coding capacity of begomoviruses, many plant factors are implicated in viral transcription and replication by interacting with virus protein or multiple *cis* elements in the intergenic region (Eagle and HanleyBowdoin, 1997; Hanley-Bowdoin et al., 2004; Lacatus and Sunter, 2009; Orozco et al., 1998). However, host factors involve in the gene transcription of begomoviruses in whitefly vectors remain unknown.

In this study, the virion-sense sequence of the right part and the complementary-sense sequence of the left part of TYLCCNV intergenic region (Fig. 1A) were used to construct the bait plasmid for yeast one-hybrid screening of whitefly proteins. We identified 51 whitefly proteins that interacted with the intergenic region of TYLCCNV (Table 1, Table 2). GO annotation analysis showed that the most of these proteins possess binding capacity and several even have transcriptional regulatory activity (Fig. S1). We then selected 32 proteins for further verification and 11 of them were confirmed to bind to TYLCCNV intergenic region (Fig. 2), suggesting possible role of these proteins in viral transcription and/or accumulation in whiteflies. Four proteins (HES1, Max, NOL11 and ADA3-A) that function in gene transcription regulation were selected for further analysis via RNAi (Griffin et al., 2015; Horiuchi et al., 1995; Iso et al., 2003; Taniguchi et al., 2016). After knocking down the expression of HES1, NOL11 or ADA3-A, the amount of viral DNA in whiteflies was decreased when compared with that of the control (Fig. 3), indicating important role of these genes in virus accumulation in whiteflies.

Mammalian HES is a homolog of *Drosophila* hairy and enhancer of split genes and plays an essential role in the development of the nervous system, sensory organism, pancreas and endocrine cells, as well as lymphocytes (Andreas and Manfred, 2007; Kobayashi and Kageyama, 2014). Besides, several studies have reported that it is involved in virus infection and degradation within host (Fan et al., 2016; Huang et al., 2017; Iwai et al., 2011; Liu et al., 2017). Li et al. (2016) found that HES could directly bind and inhibit a number of major Kaposi's sarcoma (KS)-associated herpesvirus (KSHV) lytic gene promoters. HES1 is a known transcription repressor which belongs to a family of basis helix-loop-helix proteins. However, some studies suggest that it could also acts as a transcription activator (Ju et al., 2004; Kageyama et al., 2007; Yan et al., 2002). Here we found that the whitefly HES1 binds to the consensus CACGTG motif that locates at the right part of TYLCCNV intergenic region to activate the transcription of downstream genes (Figs. 2B and 4D). Therefore, it seems that the interaction between HES1 and TYLCCNV intergenic region is essential for viral gene transcription in whiteflies. This was confirmed by our HES1 knockdown assay, both viral transcription and accumulation in whiteflies were inhibited after HES1 dsRNA treatment (Fig. 5). As a consequence of the depressed accumulation of virus in whiteflies, the virus transmission efficiency was also decreased in HES1-knockdown whiteflies (Fig. 5E).

Our results also showed that the viral DNA load in whiteflies was decreased after knocking down the expression of NOL11 and ADA3-A (Fig. 3). ADA3 is a critical component of coactivator complexes that link transcriptional activators, bound to specific promoters and basal transcriptional machinery (Saleh et al., 1997). Human ADA3 was identified as a target of high-risk human papilloma virus (HPV) 16E6 and may play a key role in HPV 16E6 related oncogenic transformation (Chand et al., 2014; Zeng et al., 2002). The relationship between virus infection and NOL11 have not been reported, however, it is localized to the nucleolus and required for pre-rRNA transcription (Griffin et al., 2015). Therefore, more investigations are needed to examine the role of these genes in virus-host (vector) interaction.

## CRediT authorship contribution statement

**Yu-Meng Wang:** Conceptualization, Methodology, Formal analysis, Investigation, Data curation, Writing - original draft, Writing - review & editing. **Ya-Zhou He:** Conceptualization, Methodology, Formal analysis, Data curation, Writing - review & editing. **Xin-Tong Ye:** Validation, Investigation. **Wen-Ze He:** Investigation. **Shu-Sheng Liu:** Conceptualization, Resources, Writing - review & editing, Project administration, Funding acquisition. **Xiao-Wei Wang:** Conceptualization, Resources, Writing - review & editing, Supervision, Project administration, Funding acquisition.

## Declaration of competing interests

The authors declare that they have no known competing financial interests or personal relationships that could have appeared to influence the work reported in this paper.
