# Supplementary material for: Whitefly HES1 binds to the intergenic region of Tomato yellow leaf curl China virus and promotes viral gene transcription
Source: Virology. 2020 Mar;542:54–62. doi: 10.1016/j.virol.2020.01.009 (PMC7031692; doi:10.1016/j.virol.2020.01.009)
Supplement: Multimedia component 1 [file mmc1.pdf]

Whitefly HES1 binds to the intergenic region of *Tomato yellow leaf curl China virus* and promotes viral gene transcription

Yu-Meng Wang, Ya-Zhou He, Xin-Tong Ye, Wen-Ze He, Shu-Sheng Liu, Xiao-Wei Wang*

Ministry of Agriculture Key Laboratory of Molecular Biology of Crops Pathogens and Insects, Institute of Insect Sciences, Zhejiang University, Hangzhou, China

* Corresponding author.

E-mail address: xwwang@zju.edu.cn (X.-W. Wang).

Supplemental information

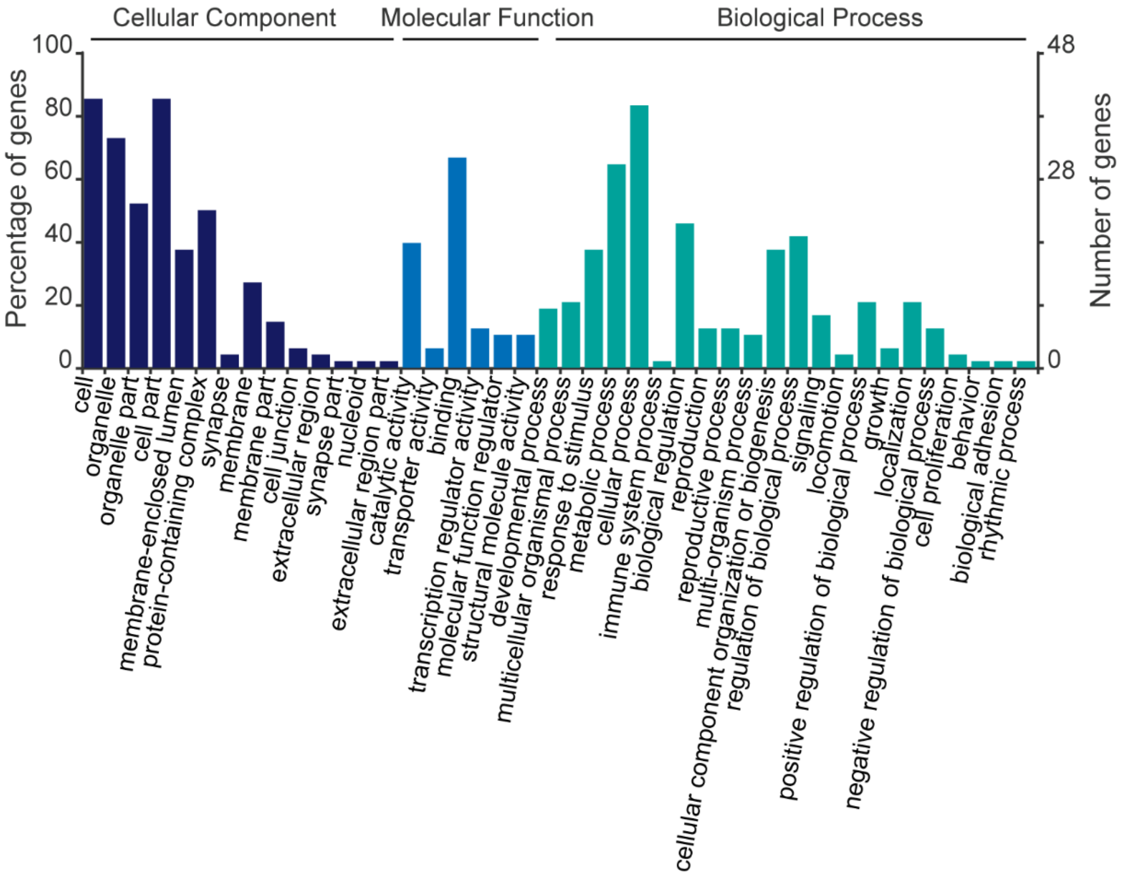

Fig S1. Gene ontology analysis of *Bemisia tabaci* proteins that interacted with TYLCCNV intergenic region in yeast one-hybrid screening. Different colors represent different GO categories.

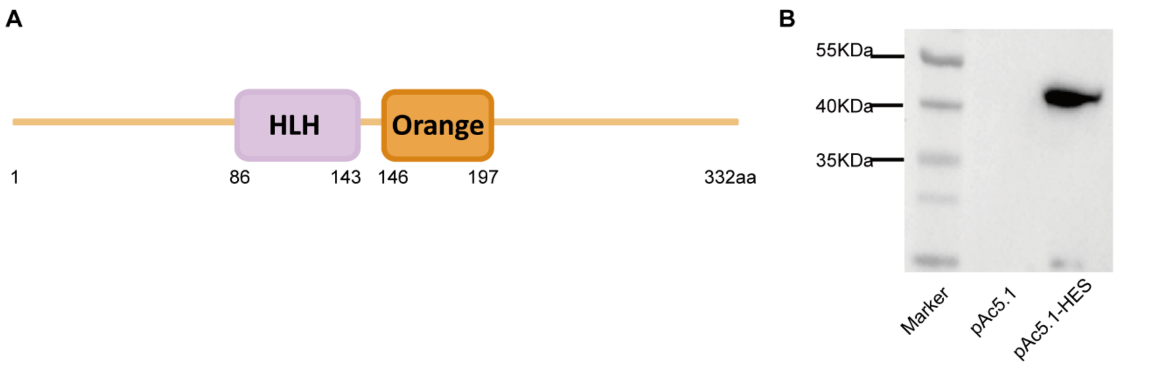

Fig S2. Predicted domains of whitefly HES1 and its expression in S2 cells. (A) Schematic diagram showing the domain composition of HES1 protein. The HLH and Orange domains of HES1 were predicted using SMART database (http://smart.embl.de/). (B) Western blot analysis of the expression of HES1 in S2 cells.

S1 Table The main primers used in this study

Primer name		Sequences(5'-3')	Length(bp)	Purpose
**TYLCCNV-Test**	F	GATATGAGATCGAAGAATCG	686bp	TYLCCNV DNA detection
	R	GCTTCGACATAATCTCTAGC		
**qTYLCCNV**	F	TTAGAGATCGTCGTCCTAGTGG	168bp	qPCR for TYLCCNV
	R	GCTCCTTACAAGCATATTGTCC		
**q-β-actin**	F	TCTTCCAGCCATCCTTCTTG	173bp	q(RT)-PCR for *β-actin*
	R	CGGTGATTTCCTTCTGCATT		
**qHES**	F	AACGAAGCACTCGAAGTTGG	114bp	qRT-PCR for *HES*
	R	TGGAAGCCGGACCTGTATTT		
**pAc5.1-HES**	F	GGGGTACCTATGACGGAGAAACTTTTGGA	999bp	Expression of HES in S2 cells (KpnI and EcoRI sites are underlined)
	R	GGAATTCCAGGGCCTCCACATCGG		
**NV-R1**	F	CCGATTTTTTTTAAAGTGGTCCCCGCAGACACGTGTGTCCAATCTTA	45bp	Construction of pGL3-basic reporter vector
	R	AGCTTAAGATTGGACACACGTGTCTGCGGGGACCACTTTAAAAAAAATCGGGTAC		
**NV-R1-M**	F	CCGATTTTTTTTAAAGTGGTCCCCGCAGAGAAATGTGTCCAATCTTA	45bp	Construction of pGL3-basic reporter vector
	R	AGCTTAAGATTGGACACATTTCTCTGCGGGGACCACTTTAAAAAAAATCGGGTAC		
**NV-R2**	F	CGGCCGCTCCTCAAAGCTTAATTGTTA	35bp	Construction of pGL3-basic

		AATGGTCCCA		reporter vector
	R	AGCTTGGGACCATTTAACAATTAAGCT TTGAGGAGCGGCCGGTAC		
**NV-R3**	F	CCTATAAAACTTAGCGCCCAAGTATTCA CGTTAAGCA	35bp	Construction of pGL3-basic reporter vector
	R	AGCTTGCTTAACGTGAATACTTGGGCG CTAAGTTTTATAGGGTAC		
**dsRNA-GFP**	F	TAATACGACTCACTATAGGGCTCGTGAC CACCCTGACCTAC	247 bp	*GFP* dsRNA synthesis
	R	TAATACGACTCACTATAGGGGTTCACCT TGATGCCGTTCTT		
**dsRNA-NLO11**	F	TAATACGACTCACTATAGGGCAGGTTAT CAGCTCAAAGAC	395bp	*NLO11* dsRNA synthesis
	R	TAATACGACTCACTATAGGGACCCTTTT GCTCAGTTTCAG		
**dsRNA-ADA3-A**	F	TAATACGACTCACTATAGGGAGTTTCTA CACCAATCGCTC	370bp	*ADA3-A*dsRNA synthesis
	R	TAATACGACTCACTATAGGGTCTTCATC ATCAGCAGTTCC		
**dsRNA-MLX**	F	TAATACGACTCACTATAGGGAGTCCTTC AGAAGTCCATTG	330bp	*MLX* dsRNA synthesis
	R	TAATACGACTCACTATAGGGACCATATC ACGCAGTTTCTG		
**dsRNA-HES**	F	TAATACGACTCACTATAGGGCAGTTCCT AGAGTCAGCTGA	421bp	*HES* dsRNA synthesis
	R	TAATACGACTCACTATAGGGAGTTCACT TGCACACACTGA		
